# Microtomographic reconstruction of mandibular defects treated with xenografts and collagen-based membranes: A pre-clinical minipig model

**DOI:** 10.4317/medoral.24811

**Published:** 2021-09-25

**Authors:** Juliana Gomez, Edmara T-P Bergamo, Nick Tovar, Huzefa S Talib, Benjamin E Pippenger, Valeria Herdia, Madison Cox, Paulo G Coelho, Lukasz Witek

**Affiliations:** 1 Department of Oral and Maxillofacial Surgery, Ascension Macomb-Oakland Hospital. Detroit, MI USA; 2 Department of Prosthodontics and Periodontology, University of Sao Paulo, Bauru School of Dentistry Bauru, SP, Brazil; 3 Department of Biomaterials and Biomimetics, New York University College of Dentistry, New York, NY USA; 4 Department of Oral and Maxillofacial Surgery, New York University, Langone Medical Center and Bellevue Hospital Center, New York, NY USA; 5 Department of Oral and Maxillofacial Surgery, New York University College of Dentistry, New York, NY USA; 6 Institut Straumann AG, Basel, Switzerland; 7 Department of Mechanical and Aerospace Engineering, New York University Tandon School of Engineering, Brooklyn, NY USA; 8 Hansjörg Wyss Department of Plastic Surgery, New York University School of Medicine, New York, NY USA; 9 Department of Biomedical Engineering, New York University Tandon School of Engineering, Brooklyn, NY USA

## Abstract

**Background:**

The goal of this study was to evaluate hard tissue response following guided bone regeneration using commercially available bovine bone grafts and collagen membranes; bilayer collagen membrane and porcine pericardium-based membrane, by means of a non-destructive three-dimensional (3D) computerized volumetric analysis following microtomography reconstruction.

**Material and Methods:**

Bone regenerative properties of various bovine bone graft materials were evaluated in the Göttingen minipig model. Two standardized intraosseous defects (15mm x 8mm x 8mm) were created bilaterally of the mandible of eighteen animals (n=72 defects). Groups were nested within the same subject and randomly distributed among the sites: (i) negative control (no graft and membrane), (ii) bovine bone graft/bilayer collagen membrane (BOB) (iii) Bio-Oss® bone graft/porcine pericardium-based membrane (BOJ) and (iv) cerabone® bone graft/porcine pericardium-based membrane (CJ). Samples were harvested at 4, 8, and 12-week time points (n=6 animal/time point). Segments were scanned using computerized microtomography (μCT) and three dimensionally reconstructed utilizing volumetric reconstruction software. Statistical analyses were performed using IBM SPSS with a significance level of 5%.

**Results:**

From a temporal perspective, tridimensional evaluation revealed gradual bone ingrowth with the presence of particulate bone grafts bridging the defect walls, and mandibular architecture preservation over time. Volumetric analysis demonstrated no significant difference between all groups at 4 weeks (*p*>0.127). At 8 and 12 weeks there was a higher percentage of new bone formation for control and CJ groups when compared to BOB and BOJ groups (*p*<0.039). The natural bovine bone graft group showed more potential for graft resorption over time relative to bovine bone graft, significantly different between 4 and 8 weeks (*p*<0.003).

**Conclusions:**

Volumetric analysis yielded a favorable mandible shape with respect to time through the beneficial balance between graft resorption/bone regenerative capacity for the natural bovine bone graft.

** Key words:**3D reconstruction, microCT, grafting material, pre-clinical model.

## Introduction

Extreme atrophy, traumatic injuries and segmental resection due to pathologies often result in anatomical, functional and physiological abnormalities of maxillofacial structures (1). Guided bone regeneration (GBR) has shown to be a promising surgical approach to restore the native osseous and soft tissue architecture, whose concept relies on defect volume filling with particulate bone graft associated with barrier membranes that allows for the osteoprogenitor cells repopulating the defect site by preventing the entry of rapidly proliferating non-osteogenic tissues (2-4). The goal of GBR procedures is to stimulate or facilitate bone regenerative potential (5). Previous studies have emphasized the advantageous ridge contour (maintenance and/or rehabilitation) for implant placement and esthetic and functional rehabilitation for GBR procedures (3-5).

The gold standard for maxillofacial bone reconstruction is an autograft, typically harvested from tibia, iliac crest, fibula and/or oral cavity, due to its exceptional osteoconductive, osteoinductive and osteogenic properties (6). While autografts prove to be advantageous, they are not without disadvantages, such as the need for a secondary site to harvest bone as well as limited quantities. Therefore a wide variety of bone substitutes, alloplastics, have been developed to facilitate with bone regeneration (6-8). Ideally, a bone substitute must be osteoconductive, osteoinductive and bioresorbable (6,7). Such assumptions associated with the recent advancements in the bone tissue engineering (BTE) have provided specific evidence to the development of several materials/techniques to regenerate bone defects including: allografts, animal-derived bone graft, synthetic biomaterials, osteogenic biomolecules such as growth factors, or a combination of techniques (1,2,5). Specifically, bovine bone mineral substitutes where organic components have been removed have been recommended to be used for guided bone formation in sinus, socket and defect augmentation procedures not only due to decreased postoperative morbidity, but also unlimited amount of available material and similarities to native human bone with respect to chemical composition and three-dimensional structure (9). Although a promising regenerative potential with favorable anatomical contour reestablishment (2,10-12), concerns have been raised according to graft remodeling/degradation rate and the possible influence on bone healing kinetics (1,2,7,11). Studies are still warranted to evaluate bone growth/graft resorption ratio to maximize regenerative potential of bone substitutes.

To improve bone healing kinetics, the use of physical barriers associated with bone substitutes are vital to provide a restrained healing scenario by preventing the migration of non-osteogenic cells, sustaining blood clot/graft in place and allowing osteoprogenitor cells to reconstruct lost tissue (3,5,13-15). Barrier membranes should fulfill four fundamental requirements: (i) biocompatibility, (ii) cellular exclusivity to avoid the defect invasion of rapid-proliferating soft tissues, (iii) favorable degradation ratio, and (iv) adequate mechanical properties to allow healing process while maintaining a space where cells from the surrounding bone tissue can migrate (5). Most recently, resorbable membranes, by using naturally-derived materials and/or employing principles of tissue engineering, have been preferentially indicated due to no need for membrane retrieval after healing, potentially increasing morbidity and adding time and cost of care (16). Specifically, collagen-based membranes have been favored over synthetic materials due to several biological properties, such as hemostatic, and cell adhesive characteristics (17-20).

Furthermore, preclinical animal models that most closely resemble human bone turnover and healing process have been the state of art concerning the evaluation of bone substitutes use in maxillofacial reconstruction (19,21-23). Usually, two-dimensional parameters of tissue regeneration are obtained by processing and quantifying serial histologic sections that represent only a fraction of the area of interest. Due to the destructive histological processing of mineralized hard tissue, which requires thick serial sectioning with a thick cutting blade, a volumetric analysis of the healing site is difficult to obtain.

The use of three-dimensional (3D) computerized microtomography reconstruction has recently gained popularity as an alternative to histomorphometry, which only approximates a fraction (~10%) of the healing site following weeks-months of processing (24). 3D reconstruction allows for the structural reconstruction of a region of interest (ROI) distinguishing materials/structures by density (25). The current *in vivo* experimental study three-dimensionally (3D) compared hard tissue response and graft degradation following guided bone regeneration procedures associating commercially-available differently-processed bovine bone xenografts (Bio-Oss® and natural bovine bone graft, cerabone®) with porcine-derived collagen membranes (bilayer collagen membrane and porcine pericardium based membrane) in minipig mandible defects and compare the results to a negative control group (no graft and no membrane).

## Material and Methods

- Particle morphology - Scanning Electron Microscopy (SEM) and microCT (μCT)

**Figure 1:**
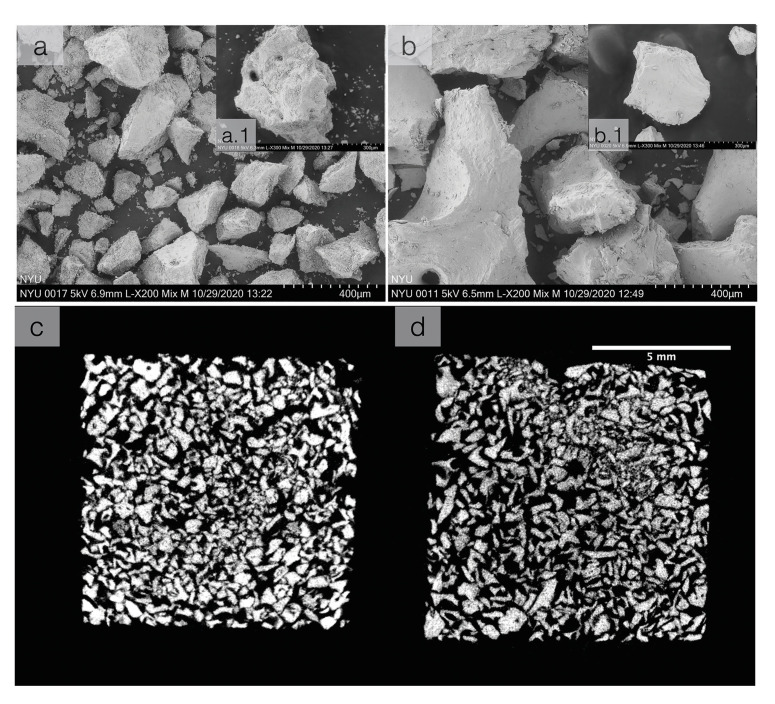
(upper) Scanning electron micrographs (SEM) of the experimental samples (a) cerabone and (b) Bio-Oss®. (Lower) 2D reconstruction slice view of the of graft material (a) cerabone and (b) Bio-Oss®, respectively.

The particulate grafting material morphology was assessed by scanning electron microscopy (Hitachi TM4000Plus, Tokyo, Japan), by spreading granules on a double-sided carbon tape. The granules were examined under an accelerating voltage of 5 kV. Bone graft samples were qualitatively assessed via SEM for surface analysis (Fig. 1). Additionally, the grafts were subjected to microcomputed tomography (μCT 40, SCANCO Medical, Bassersdorf, Switzerland). Scans were converted to DICOM files and digital data was reconstructed via 3D analysis software (Amira, Thermo Fisher Scientific, 2017, Waltham, MA) to visualize the respective grafting materials (Fig. 1).

- Experimental Design

Mandibular semi-saddle type defects, as described below, were created intraoperatively and filled with either bovine xenograft material, Bio-Oss® (Geistlich Pharma AG, Switzerland), natural bovine bone graft, cerabone® (botiss biomaterials GmbH, Zossen, Germany), or closed by primary intention without placement of material to fill the defect. Bio-Oss® material properties include heat treatment of 300°C with a particle size reported by the manufacturer to be 250-1000 μm. The natural bovine bone graft heat temperature exceeds 1200°C and the mean particle size is a narrower range of 600-900 μm as reported by the manufacturer. The porosity of Bio-Oss® is 45-63.5% compared to natural bovine bone graft which is 60-85%, both reported in literature and by the manufacturer.

The experimental defects filled with either of these materials were covered with one of two collagen membranes: fully resorbable, bilayer collagen membrane, Bio-Gide® (Geistlich Pharma AG, Switzerland), or porcine pericardium-based membrane, Jason® (botiss biomaterials GmbH, Zossen, Germany). The Bio-Gide® (approximately 1 mm in thickness) consists of one compact layer (pore size: 300 nm), which ideally prevents the infiltration of epithelial cells into the defect, approximated to a second, spongy layer (pore size: 5 μm), which ultimately allows tissue integration in the region. The Jason® membrane is very thin (0.1 - 0.2 mm) with collagen fibers organized in an opposing comb-like structure to provide tear resistance (pore size: 200-800 μm). All defects were evaluated three-dimensionally after allowing for healing time of 4-, 8- and 12-weeks and the numbers were compared to a negative control (no graft and no membrane).

- Preclinical In Vivo Model

The preclinical animal model selected for the study was the adult Göttingen minipig (Ellegaard, Denmark). Eighteen subjects, average age 22 months, were assessed and determined to be in good systemic health for use in the study. The animals were kept in designated areas under the supervision of veterinarian staff throughout the study and were allowed to acclimate for two weeks before undergoing surgical procedures.

As per the standard procedure, surgeries were performed in an animal operation suite under aseptic conditions. On the day of surgery, animal weight was obtained, and intramuscular atropine (0.05 mg/kg) was administered for premedication. Intramuscular injection of ketamine chlorate (15 mg/kg) (Ketalar Vet, Pfizer AB, Sollentuna, Sweden) and midazolam (Dormicum®, Roche, Basel, Switzerland) were administrated to induce general anesthesia. Animals were monitored to assess consciousness and once unconscious were prepared for surgery. All animals underwent surgical treatment of bilateral mandibular body; a full thickness mucoperiosteal flap was raised and atraumatic extraction of mandibular premolars (2nd, 3rd, and 4th) and first molars was conducted ensuring that the alveolar bone wall on the buccal and the lingual aspects was not damaged. This was completed by sectioning the roots in the buccolingual direction when indicated. The empty sockets were sutured with single 4-0 resorbable sutures taking care to achieve closure by primary intention (26).

The secondary surgery was conducted three months after the first stage following the same anesthesia regimen. Once unconscious, incisions were made over the crest of the bone and full thickness flaps were raised bilaterally on the buccal and lingual aspects over the edentulous areas resulting from the initial surgery. The residual ridges were flattened with a rotary bur under profuse cooling with saline. Four semi-saddle defects were created per animal, two on the right body of mandible and two on the left body of mandible. The dimensions of the created defects were approximately 15 × 8 × 8 mm (LxWxH) (~0.96 cm3 volume). The experimental groups were all nested and interpolated in each individual animal consisting of: (i) control group, no grafting and no membrane (ii) Bio-Oss® graft + Bio-Gide® membrane (BOB) (Geistlich Pharma AG, Switzerland), (iii) Bio-Oss® graft + Jason®, porcine pericardium based membrane (BOJ) (botiss biomaterials GmbH, Zossen, Germany), and (iv) cerabone® (botiss biomaterials GmbH, Zossen, Germany) + Jason® (CJ). The membranes were secured with simple tacs and single 4-0 resorbable sutures were used to close the defects. Postoperatively, antibiotics were administered for 7 days (penicillin, 20.000 UI/kg) and analgesics were administered for 3 days (ketoprofen, 1 mL/5 kg).

Animals were divided into three groups which were allowed to heal for 4 weeks, 8 weeks and 12 weeks, respectively (n=6 animals/time *in vivo*). Animal euthanasia was performed at the end of each designated healing period, and block resection of the bilateral mandible defect sites were completed with an oscillating saw to ensure that the soft tissue remained intact. All mandible samples were fixed in 10% buffered formalin solution to prepare for processing.

- Three-dimensional computerized microtomography (μCT) analysis

**Figure 2:**
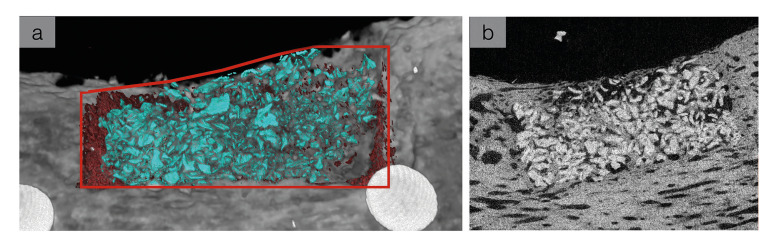
Denoting 3D reconstruction of graft material (teal) and new bone (red) overlaying original CT scan of mandible with red line border depicting the extent of defect. (b) Representative cross-sectional slice from microCT of a grafted surgical site. Graft particulates are seen with a bright opacity, relative to the native bone.

Computerized microtomography (μCT 40, Scanco Medical, Basserdorf, Switzerland) was used to obtain a 3D scan of each defect. These scans possessed resolution of 20 μm per slice with the scan conducted at x-ray energy level of 70 kVp and a current level of 114 μA. The individual files were exported to a secure server in DICOM format and imported into the 3D reconstruction software: Amira® (Mercury Computer Systems, Chelmsford, MA, USA) for reconstruction and quantification. A single experienced calibrated user utilized the software to assess the defect site in order to avoid any user volumetric rendering variation within the Amira software. Obtaining region of interest for 3D analysis was standardized for each defect, first assessing the borders of the osteotomy. The following parameters and materials were highlighted in the program: [1] borders/native bone [2] new bone [3] graft (Fig. 2), which due to their processing and respective properties, have a contrast in opacity (Fig. 2). A slice was taken virtually assessing the entire 3D structure as a 2D sagittal transection, and the exposure could be adjusted such that no noise was visible and the bone growing in from the borders was distinctly visible (Fig. 2). New bone was identified from existing bone by assessing the difference in density at the defect borders. Density of bone was subjectively assessed by the single user. Graft material appeared much denser and radio-opaque than the surrounding bone.

Once voxels (3D pixels) were assigned to each material as denoted by the user, the program calculated numerical value for each of the highlighted portions (27).

- Statistical Analysis

Percent bone formation percent and percent graft evidenced indistinguishable variances (Levene test, all *p*>0.25). These data were assessed through the linear mixed model and Tukey test for multiple comparisons with fixed factor of time *in vivo* (4-, 8- and 12-weeks) and group (control, BOB, BOJ and CJ). Eighteen individual subjects were included with two clinical defects bilaterally. Experimental sample size was 54 defects, and there were 18 control defects. All numerical data are shown as mean values with corresponding 95% confidence interval values (mean ± 95% CI). Statistical analyses of data were performed using IBM SPSS (v23, IBM Corp., Armonk, NY) setting a significance level of 5%.

## Results

All animals resumed a typical postoperative course and remained healthy without demonstrating any complications from surgical procedures. No post-operative signs of infections, inflammation and/or other clinical concerns were noted. Mandible sharp dissection after euthanasia did not reveal any clinical sign of inflammation and/or infection.

**Figure 3:**
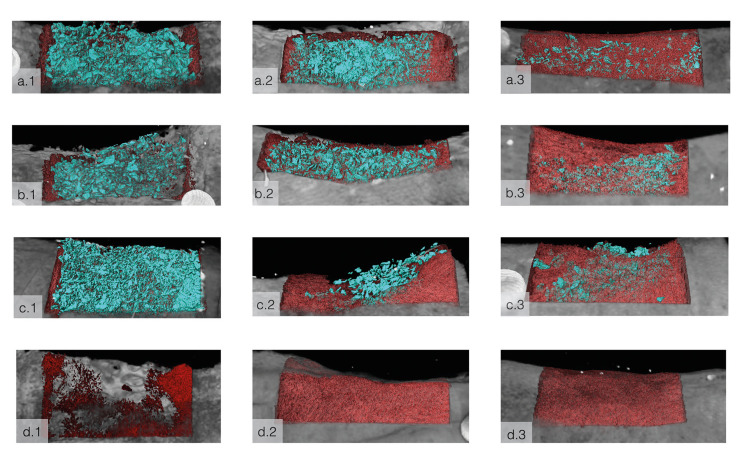
MicroCT reconstructions of (a) BioOss/BioGide (BOB), (b) BioOss/Jason (BOJ), (c) Cerabone/Jason (CJ) and d) control group; after 4-, 8-, and 12-weeks *in vivo* (columns 1, 2, and 3, respectively). Teal represents the graft material; red represents new bone in region and grey is the native bone from the original CT scan prior to reconstruction. The new bone within the defects was set to translucent for visualization of graft material.

Three-dimensional (3D) computerized microtomography (μCT) reconstruction for all time points showed gradual bone ingrowth along with the presence of particulate bone grafts bridging the defect walls for all experimental groups. New bone was shown to grow in from graft nucleation sites and from the walls towards the center of the defect for all experimental groups, and in a similar fashion from the defect walls for the negative control group. The progressive bone formation allowed for a reestablishment of mandibular bone contour for all groups (Fig. 3).

**Figure 4:**
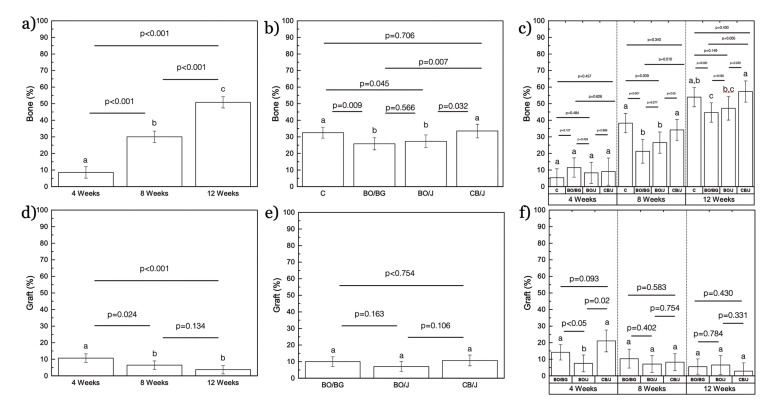
(a-c) Bone formation percent in regions of interest as a function of (a) time point, (b) group, and (c) group per time point (±95%CI). (d-f) Graft material percent as a function of (a) time point, (b) group, and (c) group per time point (±95% CI). (letters indicate statistically significant difference).

From a temporal perspective, percent new bone formation in the ROI increased from 4 to 8 weeks (*p*<0.001), and from 8 to 12 weeks *in vivo* (*p*<0.001) (data collapsed over experimental group) (Fig. 4). Percent bone formation within defect as a function of experimental group demonstrated a higher mean bone formation for Control and CJ groups compared to BOB and BOJ groups (*p*<0.045) (Fig. 4). Moreover, percent new bone formation within defect as a function of both, experimental group and time point, is presented in Fig. 4. Consistent with the aforementioned findings, no significant differences among groups in percent new bone were detected at 4 weeks *in vivo* (*p*>0.127). Statistical comparisons of Control and CJ groups demonstrated significantly higher estimated mean bone formation relative to BOB and BOJ at 8 and 12 weeks (*p*<0.039). All experimental groups presented significant increase in bone formation from 4 to 8 weeks and from 8 to 12 weeks *in vivo* (*p*<0.05).

Concerning graft degradation over time, there was a significant decrease in percent graft material present in regions of interest from 4 weeks to 8 weeks (*p*=0.024) and from 4 to 12 weeks (*p*<0.001). No significant difference was detected from 8 to 12 weeks *in vivo* (*p*=0.134) (data collapsed over experimental group) (Fig. 4). Percent graft data collapsed over time demonstrated no significant difference among all experimental groups (*p*>0.106) (Fig. 4). Percent graft material as a function of experimental group per time point is presented in Fig. 4. Despite a higher percent graft at 4-week time point for the natural bovine bone graft relative to Bio-Oss® (*p*<0.02), no significant difference was observed between graft materials at 8- and 12-week time points (*p*>0.331). Specifically, a significant reduction in the percent of graft material was observed from 4 to 8 weeks and from 8 to 12 weeks for the CJ group (*p*<0.05).

## Discussion

A predicTable outcome and reconstruction of bone defects within the maxillofacial region remains a challenge for both for surgeons and biomaterial scientist, particularly in areas where there is a need for alveolar vertical height. Adequate alveolar ridge contour favors dental implant placement in a prosthetically driven and biomechanically favorable position (28). Therefore, a substantial number of studies have focused on not only improving the surgical procedure and biomaterials that can enhance healing kinetics, but the analytical tools that can be used in predicting bone regeneration success, such as 3D volumetric analysis.

3D-volumetric analysis has the potential to non-destructively analyze the entire healing site versus the common histomorphometric analysis where substantial data is lost due to the necessary processing of mineralized hard tissue samples; A thick cutting blade is used to cut the PMMA embedded hard tissues. This can equate to analysis of only a fraction sample. To overcome the shortcomings of this type of analysis, 3D volumetric analysis was used, which can efficiently and effectively quantify the volume of graft and native bone via thresholding. It was necessary that analysis of the healing site was reviewed by a single user, minimizing variability and resulting in the proper analysis of the entire GBR site.

In the biomaterials realm, a substantial number of GBR approaches using naturally- and/or engineering-derived grafts and/or membranes have been developed (4-7). Based on such premise, the current experimental study three-dimensionally evaluated hard tissue response and graft degradation following GBR procedures associating commercially-available bovine bone xenografts (Bio-Oss® and cerabone®) with porcine-derived collagen membranes (bilayer collagen membrane and porcine pericardium based membrane) in a minipig mandible defect and compared the results to a negative control group (no graft and no membrane).

From a temporal perspective, qualitative 3D reconstruction demonstrated a gradual bone ingrowth from graft nucleation sites and from the walls towards the center of the defect for all experimental groups, and in a similar fashion from the defect walls for the control group. Quantitatively, the percent new bone formation significantly increased through all time points, which allowed for reestablishing the typical mandible shape. In fact, the periods of 4, 8, and 12 weeks *in vivo* were selected to depict tissue healing process in a timely perspective. Previous findings have demonstrated the presence of bone at different levels of maturation and physical distribution over 12 weeks. Hence, evaluating the healing process over time has the potential to allow for the identification of factors, which influence tissue regeneration and contribute to more effective future strategies.

With respect to regenerative materials, xenogeneic bone grafts are commonly used to preserve ridge contours in GBR reconstructive procedures due to their favorable biological and physical properties (e.g., similar chemical composition and three-dimensional structure to native bone) (1,10,19,22). Data analysis of the current study demonstrated higher mean bone formation for Control and natural bovine bone graft groups when compared to Bio-Oss® associated with both membranes, bilayer collagen membrane and porcine pericardium based membrane. The rationale behind the greater amount of new bone formation for the natural bovine bone graft relative to Bio-Oss® may lie primarily on graft degradation rate (29,30). In fact, the natural bovine bone graft has demonstrated more potential for rapid graft resorption over time, which has been previously shown to play a significant role in promoting osteogenesis and maximizing bone growth ability (1). Degradation rate of xenografts can vary as a result of differences in the manufacturing process (sintered or not sintered) that affect their physiochemical properties, including porosity, density, mineral ratios, and crystallinity (29,30).

Despite the current study findings corroborating the regenerative potential of bovine bone substitutes and their ability to act as a scaffold to guide new bone formation, there was no significant difference between the natural bovine bone graft, Bio-Oss® and Control groups. This fact can be associated with the dimension of the created defect, which has been previously defined as a critical factor for bone healing kinetics (16,22). Small, contained defects, as in this study, often undergo osseous regeneration without losing structural integrity and may even heal similarly to GBR augmented defects (8). The purpose of using a graft material in a small defect is to offer stability to the clot avoiding volume reduction and tissue invagination, favoring osteogenesis (2,10-12). Thus, more studies are warranted simulating the use of xenografts, mainly the natural bovine bone graft, in worse-case healing scenarios, with different defect depths and number of associated walls (9).

Furthermore, no significant difference concerning the amount of bone formation and ridge architecture preservation was observed between the two different collagen membranes (bilayer collagen membrane and porcine pericardium-based membrane). Despite structural differences with respect to membrane thickness and pore size, both seem to provide similar physical integrity that allowed for a compartmentalized healing scenario, preventing the migration of rapid-proliferating soft tissues and sustaining blood clot/graft in place (3,13-15). Moreover, their overall similar biological properties, such as the ability to attract and activate gingival fibroblast cells, periodontal ligament fibroblast cells, as well as, osteoblasts, may also be associated with the similar regenerative potential (17-20).

Surgically created maxillo-mandibular defects containing graft material with overlying membrane possess a distinct non-homogeneous pattern of bone regeneration that can be accurately assessed using a 3D analysis (25). Thus, a high resolution computerized microtomography (μCT) reconstruction has been successfully used in the present study with unequivocal advantages over two-dimensional histology, such as sample integrity and structural volumetric analysis (8,24). Nonetheless, despite the μCT reconstruction allowing for the three-dimensional assessment of the bone architecture at the created defects, clinical and histologic evaluation to confirm to what extent new tissue effectively regenerated are also required. In fact, both methodologies are important and should be simultaneously performed when characterizing naturally- and/or engineering-derived regenerative biomaterials (25).

Additionally, in terms of animal model, understanding the inherent bone characteristics associated with each species, such as bone microstructure and composition, as well as, bone modeling and remodeling properties, is important when extrapolating the results to human clinical application and should guide the selection of a preclinical study model (23). Thus, the present study selected the Göttingen minipig model that has been recognized as a remarkable model in biomedical science since its anatomical, physiological, and metabolic similarities to the human organism (21). The microstructure, bone mineral density, mineral concentration, and regeneration rate of the minipig (1.2 – 1.5 μm/day) are comparable to that of humans (1.0–1.5 μm/day). The critical size bone defect in this model resembles that of humans, the significance being such a defect that would not fully regenerate without adjunctive grafting or reconstructive techniques. This will demonstrate a bone ingrowth pattern that varies with placement of differing biomaterials in the site---which is extremely clinically applicable. These characteristics make it an ideal model to evaluate biocompatibility and hard tissue response to biomaterials prior to human use.

## Conclusions

Assessment of mandibular defects through three-dimensional computerized microtomography (μCT) reconstruction allowed for a highly accurate evaluation of hard tissue response over time. Histomorphometric analysis would have, in theory, provided for additional support of this model, however, single user-based control, standardized surgical procedure and postoperative period, and scanning protocol allowed for accurate quantification of bone ingrowth in this study. Volumetric quantification demonstrated the favorable mandible shape reconstruction over time through the beneficial balance between graft resorptive/bone regenerative capability for the natural bovine bone graft group, cerabone®. This level of osseous regeneration was comparable to the control group and higher than Bio-Oss®, irrespective of membrane type. Therefore, use of cerabone® material can be effective as an adjunctive material in the setting of larger defects as demonstrated in this study.
